# Unicentric Castleman’s Disease Presenting As Back Pain

**DOI:** 10.7759/cureus.32219

**Published:** 2022-12-05

**Authors:** Nadezda Buntic, Taylor Brown, Ummah Salma Nisar, Christopher Yeary

**Affiliations:** 1 Medicine, Lincoln Memorial University-DeBusk College of Osteopathic Medicine, Harrogate, USA; 2 Internal Medicine, Norton Community Hospital, Norton, USA; 3 Pathology, East Tennessee State University, Johnson City, USA; 4 General Surgery, Norton Community Hospital, Norton, USA

**Keywords:** flank mass, giant lymph node hyperplasia, unicentric castleman’s disease, castleman’s tumor, castleman’s disease

## Abstract

*Castleman's disease* is a rare disorder caused by a polyclonal proliferation of B lymphocytes and plasma cells. Half of all cases of multicentric Castleman's disease are associated with HIV or Kaposi's Sarcoma. Typically, unicentric Castleman's disease presents as an enlarged thoracic lymph node but can present in multiple other body areas, such as the head and neck. This case report presents a rare large extrathoracic mass causing back pain in a 71-year-old man.

## Introduction

Castleman's disease (CD), also known as giant lymph node hyperplasia, is a rare lymphoproliferative disorder diagnosed about 6600 times annually in the US [1]. There are two forms of this disease. Multicentric Castleman's disease (MCD) is a systemic disease with organ involvement that involves multiple lymph nodes. However, the more common type is unicentric Castleman's disease (UCD) which is localized to a particular region of lymph nodes. About 50% of multicentric Castleman's disease cases are found in a human herpesvirus- 8 positive (HHV-8+) individual. Most patients are immunocompromised [2]. We report a rare case of Castleman's Disease presenting as a sizeable extrathoracic mass causing back pain.

## Case presentation

**Figure 1:**
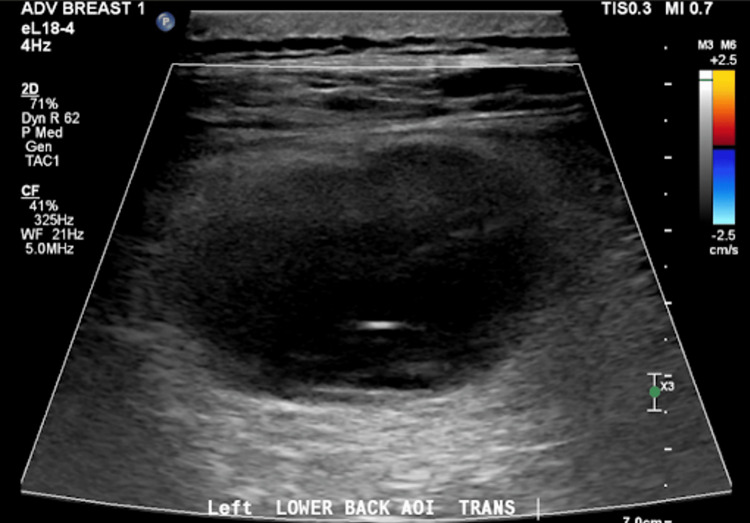
Ultrasound showing mass with complex cystic components

For the past twelve months, a 71-year-old white male was referred to the practice for a left-sided, rapidly growing flank mass. The patient endorsed intermittent back pain, 4/10, worse with movement, and located primarily on the left side. The mass was firm but non-tender to palpation, about 10cm in diameter, and non-erythematous. The borders of the mass were slightly irregular. The patient denied any neurologic deficits and was ambulatory. He denied any trauma to the area in that timeframe. He has no personal history of malignancy or familial history of lymphoma and no previous radiation to the site. He tested negative for human herpesvirus-8 and is immunocompetent. The patient had an ultrasound (Fig 1) from his primary care office before a consultation which showed a complex cystic mass with internal echoes and apparent thin septations measuring 6.3 x 5.4 x 3.5 cm.

**Figure 2:**
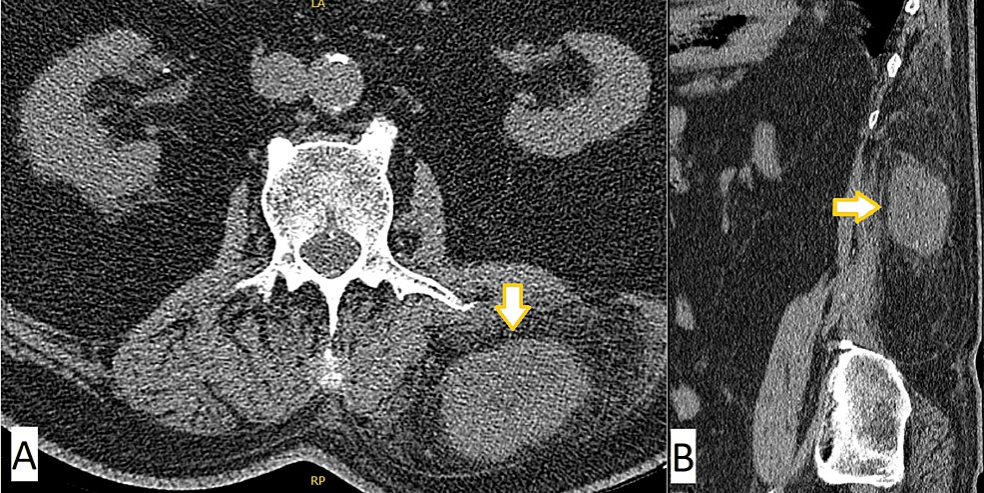
A: Axial CT image of left paraspinal mass superficial to musculature B: Sagittal CT image of mass

Given the ultrasound results and physical examination, further imaging was ordered. A CT Scan of the lumbar spine without contrast showed an isodense mass in the left paraspinal region (Fig 2). It measured 5.6cm x 3.9cm x 7.1cm, and prominent vascular structures extending to the mass were noted.

An ultrasound-guided core needle biopsy was performed with pathologic findings consistent with an intramuscular vascular proliferation with a prominent adipocytic component, which is consistent with benign vascular malformation. An excision was planned, encompassing the previous core biopsy site.

While under general anesthesia, the patient was intubated and placed in a prone position. An incision of approximately 15cm was made over the top of the lesion that reached down to the thoracolumbar fascia. The fascia was incised, with the mass noted to be immediately adjacent and adherent to the fascia. It was a yellow-white color with a rubbery feel. The mass appeared to involve the superficial ipsilateral erector spinae musculature. The mass was well vascularized, and hemostasis was achieved with metallic clips and sutures. After being removed using a Bovie electrocautery tool, the mass was handed off to the scrub tech as a pathological specimen. The site of excision was irrigated and closed with sutures. Two Jackson-Pratt drains were placed to provide bulb suction. The estimated blood loss for the procedure was 400mL. The preoperative hemoglobin was 10.3, and 26 hours postoperatively, it dropped to 8.1, prompting one unit of packed red blood cells to be transfused to the patient. He tolerated the procedure well and was discharged home on the second postoperative day.

**Figure 3:**
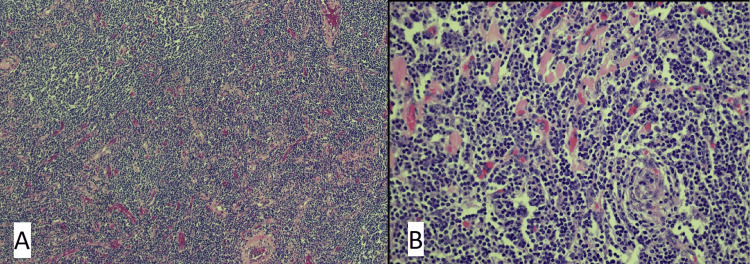
A: Pathology showing lymphoid proliferation and increased number of small vessels. Focal germinal center twinning is present (magnification x40). B: Close-up of the small vessels and onion skinning (magnification x200).

The pathological report suggested Castleman's disease, with mantle cell hyperplasia and an onion-skinning appearance of lymphocytes, which is typical for this disease (Fig 3).

HHV-8 was negative per immunohistochemical studies. CD20 highlighted B-cells were present in the slightly expanded mantle zones. No large sheets of plasma cells were present, and focal germinal center twinning was also present. These pathological findings help us conclude that this mass was likely the hyaline vascular variant of unicentric Castleman's disease. The lesion is surrounded by mature adipose tissue with no evidence of marginal involvement. After surgical resection, the patient recovered well but incidentally developed acute shingles along the left dermatome along the cephalad portion of the wound. After four weeks, the incisional pain had resolved; the patient was asymptomatic and was advised to follow up at six months.

## Discussion

Castleman's disease was first described in 1956 as a lymph node hyperplasia that resembled thymoma [3]. Two different forms (MCD and UCD) have very different presentations and should be treated accordingly. There is no gold standard for treatment. Multicentric Castleman's disease, a systemic disease involving multiple body areas, is best treated with monoclonal antibodies, such as rituximab and siltuximab [2]. Patients with MCD must continue to be monitored due to the risk of progression to lymphoma [4]. Unicentric Castleman's disease tends to stay localized to one lymph node, does not seem to require any pharmacologic treatment, and is best treated with excision [2,5]. Fine needle aspiration as a diagnosis is not indicated, as there is a risk of bleeding and a high likelihood that the amount of tissue acquired will not be enough for a definitive diagnosis [5]. The best step in UCD, as with all suspicious masses, is to send the specimen for testing after complete resection.

There are also two histological variants of Castleman's disease- the classic hyaline-vascular variant (HV) and the plasma cell variant (PC) [6]. The classic hyaline-vascular variant is characterized histologically by a prominent proliferation of vessels with hyalinized vascular walls. The plasma cell variant typically has large hyperplastic lymphoid follicles with intervening sheets of plasma cells [7].

Unicentric Castleman's disease is typically found in the mediastinum, head, neck, and retroperitoneum [3,8]. There have been reports of Castleman's disease in the epidural space, abdomen, and multiple other areas [9-10]. These cases show that Castleman's disease should be considered in a differential diagnosis for soft tissue masses and as etiologies for lymphadenopathy. 

With our patient recovering completely and resolving his symptoms, we can reasonably conclude that the cause of his pain was the mass on his back. Due to the unusual presentation of this case and the rarity of this condition, Castleman's Disease was an unexpected final pathology finding.

## Conclusions

Castleman's disease is a rare disease caused by proliferating B cells. In unicentric Castleman's disease, resection is curative and provides good results. We presented a rare case of paraspinal Castleman's disease that caused back pain. This case highlights that while rare, this disease process should be included as a differential diagnosis in abnormal soft tissue masses or lymphadenopathy.
